# Retraction Note: A fractional order nonlinear model of the love story of Layla and Majnun

**DOI:** 10.1038/s41598-024-51277-3

**Published:** 2024-01-08

**Authors:** Zulqurnain Sabir, Salem Ben Said

**Affiliations:** Department of Mathematical Sciences, College of Sciences, United Arab Emirates University, P. O. Box 15551, Al Ain, United Arab Emirates

Retraction of: *Scientific Reports* 10.1038/s41598-023-32497-5, published online 03 April 2023

The Editors have retracted this Article because of concerns regarding the originality and scientific validity of this work.

An investigation conducted after its publication confirmed that it contains material that substantially overlaps with^1^. Furthermore, concerns were raised about nonsensical content. The Editors therefore no longer have confidence in the research presented in this work.

Both Authors disagree with the retraction.
